# Genetics education program to help public health nurses improve their knowledge and enhance communities’ genetic literacy: a pilot study

**DOI:** 10.1186/s12912-021-00549-8

**Published:** 2021-02-12

**Authors:** Hiromi Kawasaki, Masahiro Kawasaki, Tomoko Iki, Ryota Matsuyama

**Affiliations:** Graduate School of Biomedical and Health Sciences, Hiroshima University, 1-2-3, Kasumi, Minami-ku, Hiroshima, 734-8553 Japan

**Keywords:** Genetics, Genetic education, Genetic counseling, Public health nurses

## Abstract

**Background:**

As human genetics knowledge develops, public genetic literacy needs to be increased, though the educational capacity for this purpose has not yet been fully developed. Under this circumstance, the daily work of public health nurses can be viewed as an opportunity to enhance public genetic literacy. However, in Japan, there is not only a lack of public knowledge of human genomics but also a lack of public health nurses’ recognition about genomic literacy. A short-term education program was implemented as a pilot study. This study aimed to examine the effectiveness of the program to support public health nurses’ activity aimed at promoting health services-related genetic literacy.

**Methods:**

The genetics education program was implemented in December 2019, in Kagoshima, Japan. Twenty-three public health nurses cooperated with the research. The program was composed of a case study on consultation, a lecture on hereditary diseases, and a discussion on the role of public health nurses. Familial hypercholesterolemia was used as the topic of the case study. We evaluated scores for cognition, affect, and psychomotor characteristics related to their learning goals before and after the program using Wilcoxon signed-rank tests. Answers in the consultation were qualitatively analyzed.

**Results:**

The mean cognitive score, capturing provision of explanations of hereditary disease, was 6.3 before the program but increased significantly to 9.3 after the program (*p* < 0.001). For the affective score, the goal of which was deepening interest in human genetics, the mean score increased significantly from 8.5 before to 11.0 after (*p* < 0.001). For the psychomotor score, addressing the need for genetic consultation, the mean score increased significantly from 4.4 before to 8.1 after (*p* < 0.001). Prominent themes extracted from descriptions on the worksheet post training included, “providing advice and accurate information on genetic disorders” and “referral to a specialized organization.”

**Conclusions:**

Our findings indicated that this education program helps public health nurses be positively involved in human genetic disorders. Thus, they may connect to their local community to provide accurate genetics knowledge and advice for health management and promoting genetic literacy.

## Background

Genetic information is currently used for diagnosis of diseases and identification of infection routes [1, 2]. However, people use genetic information and technology without acknowledging the fact that the characteristics of genetic information also include information on risks to humans (i.e., immutability, inheritance, and predictability) [3]. Genetic information can be a disadvantage for people and requires the development of an ethical code and generating public awareness of ethics to utilize genetic information [4, 5]. To help the progress of the ethical code on genetic information, the World Health Organization (WHO) proposed an ethical standard for developing medical ethics [6]. Despite the growth of the utility of genetic information for disease prevention, proper understanding [7] and use of such information is still considered insufficient. Literacy on the use of genetic information is needed.

To resolve the problem of understanding ethics and use of genetic information, it is first necessary to disseminate genetic literacy. Genetic literacy is a part of scientific literacy, and relates to the ability to use scientific thinking for personal and social purposes regarding genetics and genetic diseases [8]. Considering that prevention of health problems requires a partnership between healthcare providers and patients, both medical professionals and the general public need to have good knowledge of genetics [7]. However, this ideal state is far from the real-world situation; discrimination regarding personal genetic traits is increasingly seen (e.g., restrictions on insurance [9, 10]), and the buds of prejudice are widespread. To address problems that may result from genetic information, “genetic literacy” will be extremely important for the development of an ethical code. Even though personal genetic information was shared only by/with close relatives [11], now the general public encounters increasingly more genomic information on a daily basis [8]. They often must make decisions about whether and how to integrate new genetic technology into their lives.

To help resolve the problem of people’s inadequate understanding and ethical use of genetic information, it is necessary to disseminate educational material about genetics to the general public [8, 12]. Under conditions of low genetic literacy, the development of genetic tests and accessibility to genetic information can lead to increased discrimination and prejudice among the public. Experience of discrimination and prejudice can have a negative effect on people’s health [13]. Hence, the public must promote their understanding of genomics, the genetic diversity of humans, genetic disorders, and the rapidly developing genetic medicines based on scientific perspectives. Public education concerning genetics issues (i.e., genetic education) is now necessary to help resolve ethical issues associated with genetic technologies [14].

Genetic education for the enhancement of health literacy may be provided most effectively through school education [15]. However, genetic education was only recently introduced into the school curriculum in limited cases [16, 17]. Furthermore, by the time these children become adults, their knowledge may already be outdated. Indeed, the speed at which people improve their genetic literacy is not comparable to the speed of the development in genetic medicine and its technology. This means that the full use of genetics for disease prevention requires rapid improvements in the genetic literacy of healthcare providers, patients, and the public.

One possible way to effectively share genetic information related to their own health with the general public, is to have local health care providers (LHPs) provide the information to them [18]. Using direct contact with local people through a health care program, LHPs can play a vital role in addressing gaps in the public’s knowledge on genetics [14]. In particular, LHP public health nurses (PHNs), who usually contribute to health maintenance activities for local people through face-to-face consultations, will be best placed to provide genetics knowledge. In previous research, interviews were conducted with PHNs to assess their activities and duties related to human genetics and genetic medicine [19]. For example, some PHNs consider knowledge of genetics helps improve the health of targeted populations, and that makes them support genetics education and related healthcare services [19]. This indicates the possibility that PHNs could effectively provide genetic knowledge to a target population (i.e., contribute to “public health genetics”). However, at the same time, results of the investigation revealed an issue: PHNs rarely have opportunities to consider genetics in their daily operations. This situation means that while PHNs in principle have the potential to help others gain scientific knowledge about genetics, in practice, they do not often do it.

Additionally, there is a lack of knowledge of human genetics among many PHNs [20]. They do not have enough knowledge about genetics, nor do they have the time to prepare for genetics-related services that are in potential demand in the community [21]. The sharing of genetic information will facilitate the development of more appropriate family nursing care in genetic aspects [22]. However, in the previous example of genetic education, the link between the genetic information and nursing care was not established [23]. Even nurses caring for the symptoms of hereditary disorders find it difficult to correlate care with genetic information [22]. It is not surprising that there is a gap between genetic information and duties of PHNs, which are non-direct care duties that they had been educated on before the rapid development of genetics [24, 25].

Furthermore, in Japan, the occupational responsibilities of PHNs are legally defined by the local government. Notably, PHNs working in local municipalities are not responsible for providing social and welfare support related to genetic disorders [26]. Instead, PHNs in prefectural government, who do not usually do a face-to-face consultation with local community members unless they are diagnosed patients or highly suspected of hereditary diseases, are assigned the responsibility for genetic disorders. This may result in a situation where local PHNs overlook the potential needs of genetic knowledge and are discouraged from pursuing further studies on human genomics and associated genetic disorders. However, as noted, genetic knowledge is gradually becoming necessary for PHN work for the promotion of community health.

Training programs for genetics knowledge in human health [27] have been increasingly incorporated into basic education for nursing students, especially for PHN candidates to cope with these circumstances. However, this improvement has the same structure as medical genetics with the education of genetics for the public; this will take a long time to be effective in PHN services and contain the possibility of being outdated. Therefore, genetic training should be designed to train currently working PHNs and to help them acquire necessary skills for information and prevention vis-à-vis genetic disorders and human genetics.

In December 2019, we held a short-term pilot genetics education program for local municipal PHNs. This program aimed to share knowledge on genetics and help PHNs acquire essential skills for residents’ genetics education. To achieve this purpose, goals were set according to the three domains of learning defined by Benjamin Bloom—cognitive, affective, and psychomotor domains [28]. PHNs have fewer opportunities to provide care for genetic diseases than do staff nurses, thus their role in genetic healthcare is unclear [23]. However, PHNs have the opportunity to improve the genetic knowledge of the public. The program sought to help PHNs learn about the opportunities for disseminating genetic knowledge to the public and to facilitate genetic learning in a way that is strongly related to the duties of a PHN.

Our target population in this study were PHNs working in local municipalities, who might require basic competencies in providing genetics-related services and education to the community. The purpose of this study is to investigate the effectiveness of programs that support the activities of public health nurses to promote genetic literacy associated with health promotion.

## Methods

### Study design

The present study was designed as a semi-pilot study involving a single target group, held in Kagoshima Prefecture, Japan. Although this study was associated with a training program in facilitation skills for local municipal PHNs, where they were provided a lecture and discussed a given public health topic related to their work [29], we will not discuss the effectiveness of this event on their (general) facilitation skills. Rather, our study focused on the effectiveness of the training (the lecture and discussion regarding genetics) on the participants’ learning as it relates to human genetics.

**Fig. 1:**
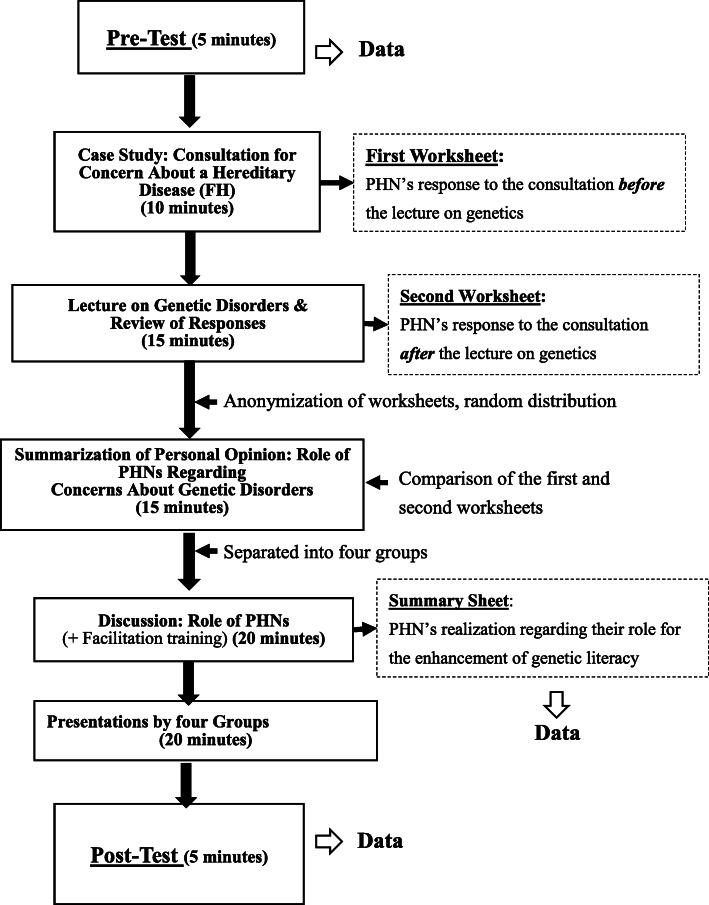
Flow of the training program and data collection

The structure of the training course is described in Fig. 1. The training was conducted so that municipal PHNs could learn the knowledge and skills needed to promote genetic literacy. The criteria for inclusion and exclusion were as follows: we included PHNs who work at the municipality office in Kagoshima Prefecture; however, we excluded the person who was in charge of providing genetic related healthcare services. Though we set the criteria above, the PHNs working at the municipality office are not usually responsible for providing the public with support for genetic diseases in the current Japanese healthcare system [19]. In fact, the participants did not include any PHNs who were involved in genetic diseases. The training was designed based on a similar trial, which aimed to support the decision-making process of local people through consultation on their genetics-related issues [29]. The main results were obtained by the measurement of the participants’ cognition, affect, and psychomotor characteristics, (described later) before the training (pre-test) and again after the training (post-test). In addition, participant’s thoughts on the role of PHNs in genetics consultation, based on the description in the summary sheets provided in their discussions, were analyzed. Details of the intervention are given in “Training for PHN participants.”

### Training for PHN participants

The motivation for professionals to learn is increased when learning is associated with their work [30]. To motivate learning about genomics for PHNs, who are not responsible for providing support to people diagnosed with genomics disorders in their daily work, we used lifestyle-related disease case studies as skills training for the participants to learn about genomics. Residents who have not been diagnosed with a genetic disease may still have a genetic disease. Through learning basic counseling skills, we provided the minimum genomic knowledge necessary for the case. The training was limited in that it only addressed genetic knowledge relevant for counseling one genetic case study.

First, a workshop with a case study was held to measure whether the knowledge of human genetics and related disorders was linked to the daily consultation of PHNs. In this workshop, familial hypercholesterolemia (FH) was selected as a topic of consultation from a simulated client (i.e., the PHN was asked whether hypercholesterolemia is hereditary, see “Details of Case Study”). FH is an inherited autosomal disorder of lipid metabolisms. When the gene encoding the low-density lipoprotein (LDL) receptor in chromosome 19 has double abnormal copies (homozygote), the rate of atherosclerosis accelerates from childhood. Although the prevalence of homozygous FH is not very high (about one in a million), it is a “designated intractable disease” in Japan [31]. Although the proportion of patients with FH is small, such patients are entitled to various forms of public support, and the disease is important for PHNs because support is typically provided by prefectural PHNs through consultation with patients and confirmation of diagnostic evidence such as a genetic test. It is the role of the PHN of the municipality to support the residents until they are connected to the PHN of the prefecture. In addition, in cases where the gene related to FH has only one abnormal copy (heterozygote), it still presents a genetic risk for the onset of atherosclerosis [32]. Though the incidence of the atherosclerosis in heterozygous FH patients is similar to those of dietary or age-related dyslipidemia, knowing the genetic information of the patient and his/her family will be important for PHNs. Specifically, it provides an opportunity for PHNs to help guide the patient to improve his/her life at an early stage.

After introducing this simulation example, participants were asked to write a scenario on a worksheet (the first worksheet) describing how they would respond to local people on this topic. These responses were used as a resource for discussions about the role of PHNs for informing community members about human genetic disorders.

Second, a lecture was delivered for 15 min. Our education materials gave (i) information on basic health promotion topics selected from materials related to genetics education; genetic factors and environmental relations; (ii) general information needed to understand human genetics and genetic disorders; human genome as the blueprint for life; characteristics of genetic information: immutability, predictability, and commonality; and (iii) detailed information about multifactorial genetic disorders that have been believed to be caused by patients’ personal body constitution and not by their genetic background; diabetes, dyslipidemia, hyperlipidemia, and FH were used as examples. All of those diseases are common disorders related to PHNs’ daily operations that admit possible causation by genetic factors. After the lecture, to encourage participants to review their responses to the consultation in the case study, a second worksheet was distributed, and the participants were asked again to describe how they would respond to the consultation.

Following the lectures and review of the case study with the PHNs, time was provided to participants to consider the role of PHNs regarding genetic disorders. In this process, we anonymized the worksheets collected in the case studies and randomly distributed them to the participants as materials for consideration; participants critically reviewed the descriptions by comparing the first and second worksheets that other participants had completed. This randomization was meant to prevent the participants from experiencing any shyness about their lack of knowledge and/or indifference to genetic disorders written on their own first worksheet. Through this process, participants summarized their personal opinions to the target of interest, then prepared for the subsequent discussion.

In the discussion, participants were divided into four groups and debated the role of PHNs in consultation regarding genetic disorders. Through this process, the perceived role of PHNs was extracted and written on the summary sheet (Fig. 1). This summarization task was also aimed at helping participants learn how to facilitate relevant genetic discussion during a consultation. Finally, each of the four groups provided a presentation of their summary.

### Details of case study

The case study used in the training included a simulation scenario in which a member of the community had raised concerns about FH during a health check-up. This person was a 40-year-old man who lived with his wife and 18-month-old son. He was taking medications for hyperlipidemia, and his wife was aware of this. He reported, “My father passed away from myocardial infarction at the age of 55. I also know my grandfather died from heart disease. I am worried that I may have inherited the same condition.”

### Data collection

The attainment goals of the training were set according to each of the three domains of learning defined by Benjamin Bloom—the cognitive domain, the affective domain, and the psychomotor domain [28]. In our genetics education program, the target for the *cognitive domain* was set as the participants become able to explain genomic diseases; for the *affective domain,* the participants become interested in human genetics; and for the *psychomotor domain,* the participants become able to address the needs of community members by using their knowledge of genetics. To quantify the degree of achievement, we extracted the data from the worksheet and the survey forms through the following processes. An ID was assigned to each participant and used throughout the training session, written on the survey forms. Survey results from before and after the training were linked at the individual level and then compared using the assigned IDs. By extracting personal responses from the worksheets and corresponding post-discussion feedback, we were able to examine the degree to which participants achieved their goals in genetics literacy.

### Evaluation of training

#### Data analysis

The effect of training was evaluated by comparing survey scores from before and after the training session. The items were rated using a 5-point Likert-type scale. A Wilcoxon signed-rank test was performed to compare the significance in differences of scores between the pre-test (before the lecture and the workshop) and post-test (after the lecture and the workshop). To evaluate the difference in attitudes toward human genetics by PHNs in the current situation (i.e., under the absence of genetics education), associations between age groups and scores in pre-test and post-test, and between years of experience and scores in pre-test and post-test, results were quantified by Spearman’s ρ. The statistical analyses were carried out using SPSS (v. 25) and R (ver. 3.6.2) [33], with a significance level consistently set as 0.05. The PHNs’ responses and reactions to the simulation were extracted from the descriptions given in the worksheets during the workshop. Descriptive and qualitative analyses were performed. Specifically. we adopted the thematic analysis method recommended in the literature [34]. For all analyses, we extracted specific phrases corresponding to each learning domain. Hereafter, the extracted phrases are indicated in square brackets (i.e., []), and the original texts and codes are shown in single quotation marks (i.e., “). The specific phrases were classified into categories based on their semantic content. The achievement of the goal was verified for each category.

#### Indices for evaluation

Subordinate questions were created for each domain (i.e., cognitive, affective, and psychomotor) to evaluate the degree of achievement for each attainment goal. These items were created based on a previous study [29] using a 5-point Likert-type scale. All questions were positively orientated: ‘Not at all applicable’ was 1, ‘Slightly applicable’ was 2, ‘Somewhat applicable’ was 3, ‘Applicable’ was 4, and ‘Very applicable’ was 5. Since each domain had three subordinate questions, the maximum score for each domain was set as 15 (and the maximum total score provided by the three domains was 45).

Subordinate questions for the ‘Cognitive domain: Ability to explain genomic disease’ are as follows:

- I am familiar with the term “human genomics.”
- I can explain diabetes by referring to hereditary and environmental factors.
- I have had the opportunity to get accurate information about hereditary diseases. (^†^Note that we used an example of diabetes as genetic related diseases as well as FH in the lecture of the training course.)

Subordinate questions for the ‘Affective domain: An interest in people’s genetics’ are shown below.

- I am interested in studying human genetics.
- I have wanted to obtain accurate information on genetic disorders.
- I am interested in news and articles related to human genetics.

Subordinate questions for the ‘Psychomotor domain: Ability to address the needs of local people by using knowledge about genetics’ are shown below.

- I can fully explain human diversity using genomic information.
- I can proactively study and consider human genetics by myself.
- I can respond to concerns raised by a member of the community by using knowledge of genetics.

### Ethical considerations

Our study was approved by the research ethics review committee of the Hiroshima University (approval number: E-1776-1). Our scope of analysis was limited to participants who had completed the training, that is, the PHNs who attended the training course, which was organized by a PHN group consisting of nurses working in local municipalities. Both the organizer and the participants of the session provided written consent to participate in this study. No reward was offered to participants.

## Results

### Overview of participants

There were 36 PHNs working in this area. Training for the duties of PHNs is provided by municipalities within the same jurisdiction. Twenty-six people were able to participate in the training program without having their other duties affected.

Our participants included 23 PHNs who answered surveys and provided their written informed consent to participate. All participants were women, and the average years of working experience as a PHN were 11.9, ranging from one to 35 years. Of all the PHNs, only three had experience in genetics-related consultations (Table 1). Two were in their 20s, and one was in their 30s.

**Table 1:** Summary of participants

Variable	Category	Number of Participants	%
Age group	20s	7	30.4
	30s	7	30.4
	40s	7	30.4
	50s	2	8.8
Experience of a consultation on genetic disorders	Yes	3	13.0
	No	20	87.0

### Evaluation of associations between scores, age groups, and years of experience

In the pre-test, we found a significant negative correlation (*ρ* = − 0.461, *p* = 0.027) between participants’ age group and the first cognitive question, ‘I am familiar with the term “human genomics”.’ Answers to all the remaining questions were correlated with years of experience. For the relationships between age group and the score on each domain (cognitive, affective, psychomotor), and the sum of scores of the three domains (total score), the results of the correlation analyses are shown in Table 2. All the coefficients show significantly negative associations except for the psychomotor score. These results show that younger PHNs have more frequently heard the word “genome” and have higher cognition, psychomotor, and total scores than older PHNs. On the other hand, there was no significant association found between the age group and questions, scores, and sum of scores on the three domains in the post-test.

**Table 2:** Correlation between age group and scores before learning about human genetics

Domains	Age group	
	coefficients	*p*-values
Cognition	−0.397*	0.021
Affective	−0.225	0.193
Psychomotor	−0.384*	0.035
Total	−0.342*	0.042

*p< 0.05

Although the coefficients were not significant for affective and total scores, similar correlations were observed between the scores and age (Table 2). In the post-test, there was no significant association found between the years of experience and questions, scores, and sum of scores of the three domains.

### Difference in sum of the scores observed in the three domains

**Fig. 2:**
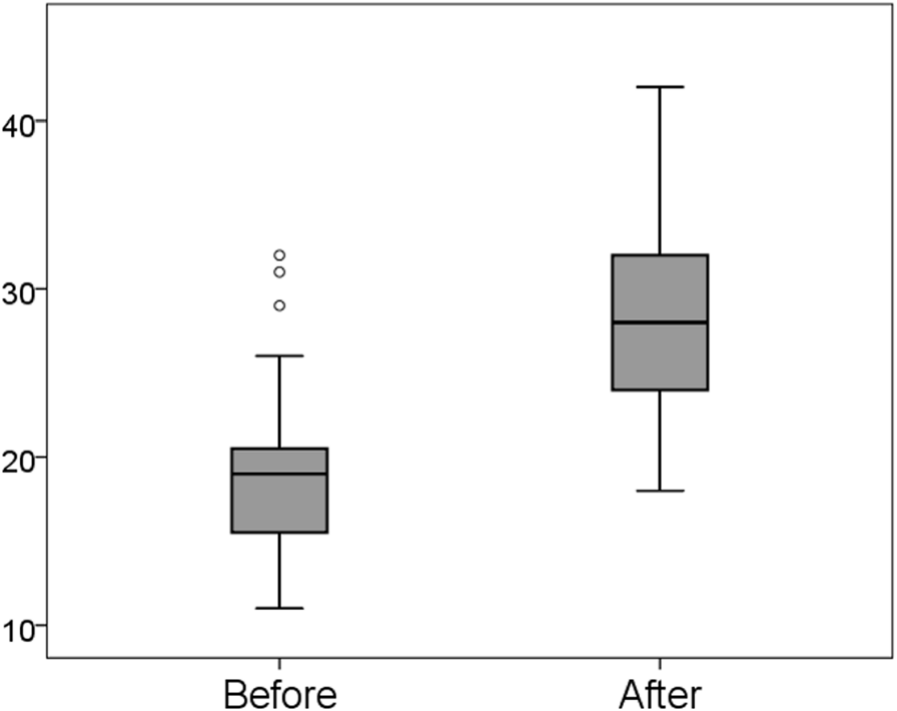
Changes in all scores from before to after training. FH: familial hypercholesterolemia; PHNs: public health nurses, LHPs: local health care providers

The mean score of the sum of the three domains was 19.3 (SD = 5.57) before training and 28.5 (SD = 6.40) after training. The Wilcoxon signed-rank test showed that the distribution of total scores was significantly different before vs. after training (*p* < 0.001; See Fig. 2).

### Difference in scores on the cognitive domain

**Fig. 3:**
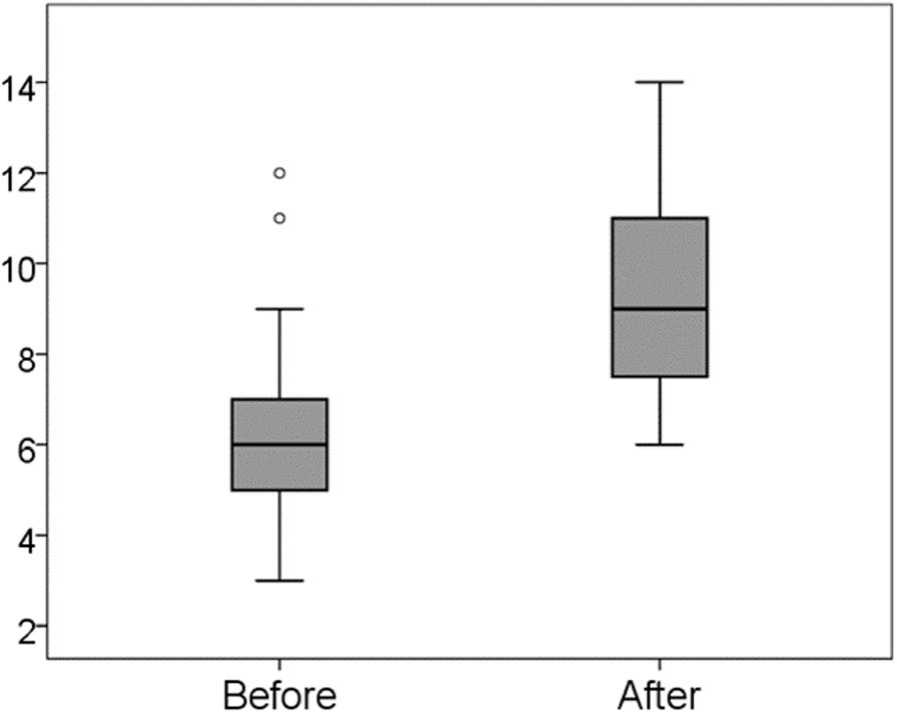
Changes in the cognitive domain score from before to after training

As shown in Fig. 3, the mean score on the cognitive domain significantly increased from 6.3 (SD = 2.34) before to 9.3 (SD = 2.32) after training. Wilcoxon signed-rank test demonstrated a significant difference between those distributions (*p* < 0.001).

### Difference in scores on the affective domain

**Fig. 4:**
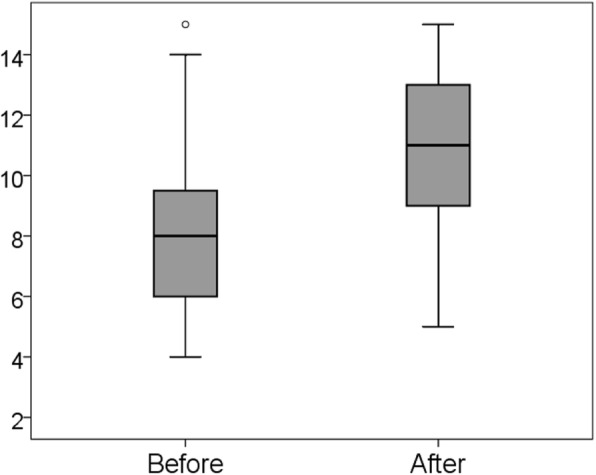
Changes in affective domain score from before to after training

As shown in Fig. 4, the mean score for the affective domain also significantly increased, from 8.5 (SD = 2.71) to 11.0 (SD = 2.64; Wilcoxon signed-rank test, *p* < 0.001).

### Difference in scores on the psychomotor domain

**Fig. 5:**
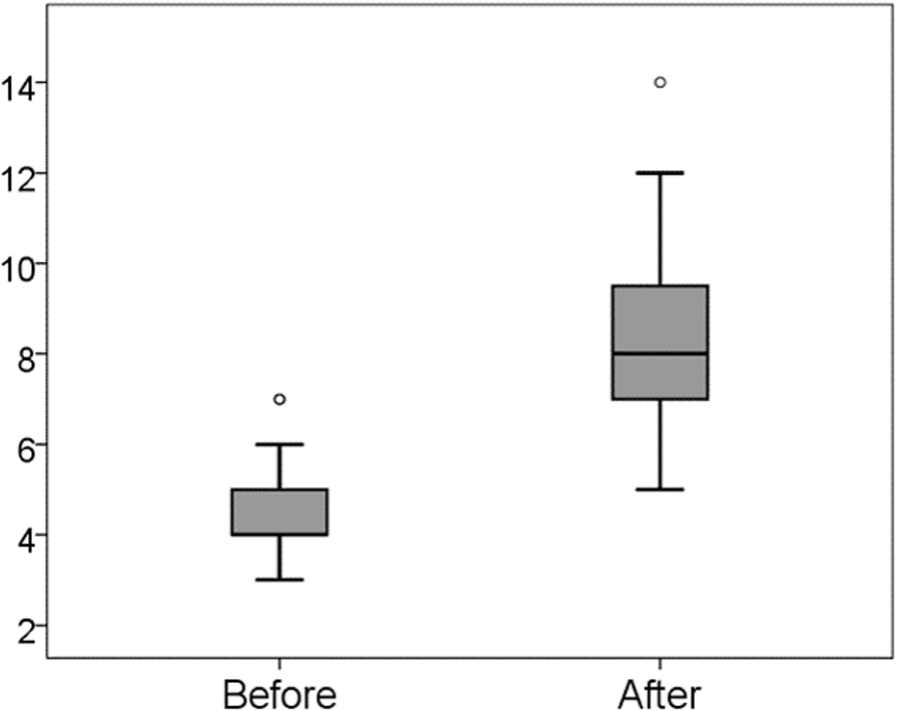
Changes in the psychomotor domain score from before to after training

As in the cases of the two previous topics, the mean score for the psychomotor domain also significantly increased from 4.4 (SD = 1.12) to 8.1 (SD = 2.39; Wilcoxon signed-rank test, *p* < 0.001) (Fig. 5). On the third question, ‘Can you respond to concerns raised by a member of the community by using your knowledge of genetics?’ 10 of 23 participants reported they could after training and none before the training.

### Descriptions of responses during consultation

Participants wrote their thoughts on genetics, which were then extracted from the worksheets. Regarding their roles in genetics education to the community, [providing advice thought to be potentially required for individual life] and [providing people with accurate information about genetics] were found often (Table 3).

**Table 3:** Participants’ responses during the consultation

Categories	Code of responses during consultation
Extract problems with genetic disorders	Summarize the anxiety
	Check the facts about genetic disorders
	Organize by listening to the necessary narratives of the person
	Share your concerns about heredity disorders
	Ask why they thought they inherited the disorder
	Gather information for identifying the genetic disorder
	Determine if the person’s troubles are hidden
	Confirm the fact that you thought it was inherited
	Confirm knowledge about illness
	Listen to their understanding of the disease
	Pay attention to their family background and life
	Accept anxiety about getting the same disease
	Ask about their lifestyle
	Ask about their life
	Pay attention to and manage previous living conditions
	Indicate the problem
	Make sure that you express you are very worried^a^
	Identify problems from statements and appearances
	Determine issues based on information and prioritize
	Ask what the person wants to do in the future
	Promote awareness of other causes of health deterioration
	Pay attention to medical condition/treatment
	Check the process until the start of treatment
	Paying attention to the treatment status and changes toward problem solving
	Collect information about their health
Providing advice and thoughts potentially required for individual life	Suggest a countermeasure
	Thinking together while advising the person on what they think is necessary
	Do not get preoccupied with hereditary conditions
	Lead a safe life
	Check what can be advised
	Health guidance for life improvement
	Health guidance closely related to daily life
	Provide information about what you can do
	Promote health behaviors such as undergoing regular medical checkups
	Create an opportunity to appraise your lifestyle
	Appraise lifestyle habits other than heredity-related aspects
	Create an opportunity to appraise patient’s lifestyle
	Thinking together about ways to improve the situation
	Encourage the patient to notice the problem
	Notice the problem^b^
	Suggest a solution based on what you can do
	Suggest what could be improved
Providing people with accurate information about genetics	Assess the risk of onset
	Thinking about ways to reduce the risk of onset
	Providing the correct information
	Providing expert knowledge to reduce anxiety
	Explain the relationship between lifestyle and heredity
	Tell the possibility of inheritance
	Respect the will of the other person
	Explain potential genetic effects
	Explain environmental factors
	Suggest preventive lifestyle behaviors
	Explain precautions in daily life and help prevent onset
	Informing the person of potential genetic risks
	Suggest what can be improved to delay the onset
	Give an explanation that can be understood by the other person
Introducing the person to an appropriate specialized organization	Introduce them to a specialized institution
	Introduce the person to doctors and other necessary healthcare institutions and healthcare professionals
	Introduce the person to a professional consultation agency

^a^ The consultant was asked to express that they are very worried

^b^ The consultant was asked to identify the problem

In our qualitative interpretation of extracted phrases, we found that a phrase ‘testing is needed to determine if your disease is inherited, let me introduce you to an expert on human genetics’ was reflected on the PHN’s role of [introducing the person to an appropriate special organization]. Moreover, the phrase ‘although the body constitution is inherited, you will not necessarily become ill’ indicated that the participants recognized that the onset of the disease is related not only to physical constitution as a manifestation of personal genetic traits, but also to their lifestyle; therefore, an ‘explanation of environmental factors is needed’; that is, [providing people with accurate information about genetics]. This extracted phrase includes ‘tell the possibility of inheritance’ and ‘explain the risk of development of diseases.’ Participants had a deeper understanding of their role of providing care for genetic disorders as PHNs.

## Discussion

We implemented a short-term pilot education program with an aim of helping PHNs working for local municipalities enhance their knowledge of human genetic disorders and provide better genetics-related advice and services to community members, as this is an important role bestowed upon them. Bearing in mind that the effectiveness of our genetics education was measured in a self-report style by the participants, their cognitive, affective, and psychomotor domains related to the learning of public health genetics were significantly improved by the training program; that is, the program was at least effective in the short term for learning about public health genetics by PHNs. In the pre-test, a trend was seen where young, relatively less experienced PHNs scored higher on the cognitive and psychomotor domains, and the sum of all three domains was higher, indicating that the scores may also depend on the content of the educational curriculum. However, this trend disappeared in the post-test. In addition, from a descriptive analysis perspective, the training clarified that the participants’ understanding of the importance of their role in genetics education for local communities has deepened, as seen in the extracted phrases relating to informing about genetic risk provided by inherited bodily constitutions, the existence of genetic testing, and expert persons.

Previous studies have indicated that PHNs working for local municipalities do not perceive hereditary diseases as genetic disorders caused by defective genes, but as a phenomenon caused by environmental and social factors shared among members of the community and family [19]. In fact, many previous studies have found that risk of cardiometabolic diseases (e.g., obesity, hypertension) varies depending on the geographical area [24, 35], socioeconomic status [36], and environment [37]; in other words, members from the same community may be exposed to the same environmental and/or socioeconomic factors due to living in the same area and abiding by the same culture and lifestyle, and may even develop the same diseases. Consequently, even if concerns are potentially related to genetics (i.e., heredity and family history), PHNs might not perceive consulted symptoms as genetic disorders per se, but rather as products of complex environmental and socioeconomic conditions. Regardless, through the present study, the need for genetic knowledge and subsequent genetic consultation by PHNs was clarified; some young PHNs stated they had experienced consultations regarding genetic concerns from members of their target population. As seen in the responses after the discussion, not only among younger PHNs, participants have noticed that the genetic aspects of healthcare advice need to be covered in addition to environmental and socioeconomic factors; furthermore, that this will provide a clearer and more reasonable explanation of disease risks than that provided at current consultations. This was similar to an expected phenomenon described by Tonkin et al. (2011) [38]: deepening knowledge of genetic disorders will help in understanding why certain people are more likely to develop certain diseases [38]. Their awareness shows that, as we expected, the training was effective. This educational program was considered, to some extent, effective in PHNs for the understanding of genetic disorders as causes of diseases and also for increasing motivation to learn about them.

While there are many online resources for learning about human genomics and genetic disorders [38], PHNs seem to be facing difficulty allocating the time to study the subject [39]. This was seen in their comparably low scores on cognitions and affect domains in the pre-test, and possibly resulted in the current situation where they have had limited opportunities to learn about human genetics in their daily occupational life. It is well known that adults’ motivation to learn new skills or attain new knowledge generally needs to be strongly related to their interests or profession [30]. To address this problem, we propose that introductory learning in genomics must be incorporated into work-related training. It means that cases of genetic disorders are used as teaching materials to develop the PHN’s standard “career ladder” for local governments [40, 41]. We suggest conducting routine training courses of introductory-level genetics, such as the pilot programs used in the present study, and continuously providing follow-up education programs utilizing accessible sources, such as web-based material [41]. If it is organized by professional decision-making bodies (e.g., local, prefectural, and national government, or associations and/or organizations related to PHN) and widely recognized as a required professional skill, we think it will assist PHNs in trying to develop essential technical skills.

The scores of all three domains increased in the post-test. In particular, there was an increase in the number of PHNs who indicated that they could handle a consultation with a member of the community (as seen in the third question of the psychomotor domain). Participants’ responses during consultation indicated that the consultation on genetics has also changed to activities based on the duties of a PHN. There is a need to apply counseling techniques to identify problems related to genetic disorders, conduct health assessments, and provide appropriate advice to manage identified problems. The goals of the psychomotor domain can be achieved by incorporating them into PHNs’ regular duties. For some genetic disorders, the risk of onset can be assessed by genetic testing. Further, onset of symptoms can be delayed and managed by lifestyle changes. The utilization of knowledge relating to human genetics is desired in public health services for disease prevention and health management, and may enable reduction of healthcare costs [42, 43]; good effects have already been seen as a result of screening of some genetic disorders [44] and genetic testing for Alzheimer’s disease [45]. The results of our short-term education program suggested that it may also indirectly contribute to those ethical aspects. Certainly, these conclusions may be inflated because we only assessed short-term enhancement of the responses of PHN for public health genetics.

The study has a few limitations: (i) we could not measure the long-term effect of our education program, (ii) the number of participants was limited to 23, and (iii) the effect of education was measured only by self-reported questionnaires. For (i) and (ii), in future research, we should design a program that will give us the capability to assess long-term effects. Considering the effect of training for radiation education, Japanese PHNs who were not in charge of the consultation regarding radiation hazards maintained the education effect 1 month after the training [29]. It was suggested that PHNs who recognize the relationship between their duties and the problems (i.e., here, the radiation hazard) are easily motivated to learn about the problems [46–48]. Moreover, PHNs in multiple locations and with different cultural backgrounds should be recruited. For (iii), the development of methodologies that objectively measure knowledge level, the capability of consultation, and the motivation of participants relating to public health genetics are required. Perhaps, knowledge tests, role-play, and evaluation from third party members will be helpful tools.

By implementing a simple case study, we provided an opportunity for PHNs to consider their role relating the informing and education of genetic disorders to local communities. PHNs who participated in our study described their role in public health genetics as “providing advice and accurate information to targeted individuals, and referral to a specialized organization.” The target population of this study was only a limited number of PHNs from a limited number of local municipalities. However, as mentioned above, we think this program should be tried by many on-site PHNs working within different cultural backgrounds. Depending on local characteristics, the familiar cases of genetic disorders used in the teaching material may change. Nevertheless, we believe this type of training will motivate PHNs to learn more about human genomics and genetic disorders, increase their genetic literacy, and thereby also improve public literacy. This will contribute to the effective implementation of genetic testing for the early detection of disease risk.

## Conclusions

A short-term pilot education program regarding human genetics was conducted for PHNs at a local municipality in Japan. The results of our program demonstrated the achievement of the following goals: first, helping PHNs gain basic knowledge and develop a deeper interest in human genomics and associated genetic disorders; and second, helping PHNs understand their role and the possible opportunities for providing local people with accurate knowledge of human genetics. Bearing in mind that this is a short-term program with a limited sample size, future research should consider implementing this program over the long-term, among PHNs working with people of different cultural backgrounds. Certainly, political amendments by lobbying healthcare and political leaders are needed to change the current situation of nursing practice. This training could potentially motivate more PHNs to learn more about genetics, which may enhance their knowledge and thought process related to genetics, and thereby prepare them to provide care for target residents. Thus, we believe this program can contribute to the enhancement of the use of genetic testing and subsequent early detection of disease risk by improving the genetic literacy of local communities through the services provided by local PHNs.

## Data Availability

The datasets generated and/or analyzed during the current study are not publicly available due to the need to maintain anonymity of participants and the confidentiality of the data. However, the datasets are available from the corresponding author on reasonable request.

## Abbreviations

WHO: World Health Organization
UNESCO: United Nations Educational, Scientific and Cultural Organization
LHPs: Local health care providers
PHNs: LHP public health nurses
FH: Familial hypercholesterolemia

## References

[1] Genetic Testing Registry [Home page on the Internet]. https://www.ncbi.nlm.nih.gov/gtr/. Accessed 11 Nov 2020.

[2] Dos Santos WG (2020). Natural history of COVID-19 and current knowledge on treatment therapeutic options. Biomed Pharmacother.

[3] The Japanese Association of Medical Sciences (2011). Guidelines for Genetic Tests and Diagnoses in Medical Practice.

[4] Evans M, Mathews AW. Doctors limit what to tell patients about their DNA test. Should they? The wall street Journal, 4 October 2019. https://www.wsj.com/articles/doctors-decide-what-to-tell-patients-about-their-dna-test-should-they-11570202161. Accessed 24 Nov, 2020.

[5] Hill WD, Davies NM, Ritchie SJ, Skene NG, Bryois J, Bell S (2019). Genome-wide analysis identifies molecular systems and 149 genetic loci associated with income. Nat Commun.

[6] World Health Organization (1998). Proposed international guidelines on ethical issues in medical genetics and genetic services.

[7] Boerwinkel DJ, Yarden A, Waarlo AJ (2017). Reaching a consensus on the definition of genetic literacy that is required from a twenty-first-century citizen. Sci & Educ.

[8] Chapman R, Likhanov M, Selita F, Zakharov I, Smith-Woolley E, Kovas Y (2019). New literacy challenge for the twenty-first century: genetic knowledge is poor even among well educated. J Commun Genet.

[9] Billings PR, Kohn MA, de Cuevas M, Beckwith J, Alper JS. Discrimination as a consequence of genetic testing. Am J Hum Genet 1992;50:476–482. PMC1684266 1539589

[10] Bélisle-Pipon J, Vayena E, Green RC, Cohen IG (2019). Genetic testing, insurance discrimination and medical research: what the United States can learn from peer countries. Nat Med.

[11] Bereshneh AH, Nejad AS, Akrami SM (2015). Genetic counseling and genetic tests ethical challenges. J Clin Res Bioeth.

[12] Abrams LR, McBride CM, Hooker GW, Cappella JM, Koehly LM (2015). The many facets of genetic literacy: assessing the scalability of multiple measures for broad use in survey research. PLoS One.

[13] Pascoe EA, Smart RL (2009). Perceived discrimination and health: a meta-analytic review. Psychol Bull.

[14] World Health Organization (2003). Review of ethical issues in medical genetics.

[15] Institute of Medicine (US) Committee on Assessing Genetic Risks (1994). Public education in genetics assessing genetic risks: implications for health and social policy.

[16] Hicks MA, Cline RJ, Trepanier MA (2014). Reaching future scientists, consumers, & citizens: what do secondary school textbooks say about genomics & its impact on health?. Am Biol Teach.

[17] Kohama N, Kawasaki H, Kukinaka C, Goda H, Rahman MM (2020). Identifying the challenges to successfully teaching about genetic diversity among Japanese junior high school students. SAGE Open Med.

[18] Syurina EV, Brankovic I, Probst-Hensch N, Brand A (2011). Genome-based health literacy: a new challenge for public health genomics. Public Health Genom..

[19] Goda H, Kawasaki H, Masuoka Y, Kohama N, Rahman MM (2019). Opportunities and challenges of integrating genetics education about human diversity into public health nurses’ responsibilities in Japan. BMC Nurs.

[20] Birks M, Ralph N, Cant R, Hillman E, Chun TY (2015). Teaching science content in nursing programs in Australia: a cross-sectional survey of academics. BMC Nurs.

[21] Sasaki N, Morifuji K, Tsubota S, Matsumoto T, Miyahara H (2015). Experiences of public health nurses providing genetic counseling in underpopulated areas of Nagasaki Prefecture. J Jpn Soc Genet Nurs.

[22] Calzone KA, Cashion A, Feetham S, Jenkins J, Prows CA, Williams JK (2010). Nurses transforming health care using genetics and genomics. Nurs Outlook.

[23] Godino L, Turchetti D, Skirton H (2013). Knowledge of genetics and the role of the nurse in genetic health care: a survey of Italian nurses. J Adv Nurs.

[24] Daniel CR, Prabhakaran D, Kapur K, Graubard BI, Devasenapathy N, Ramakrishnan L (2011). A cross-sectional investigation of regional patterns of diet and cardio-metabolic risk in India. Nutr J.

[25] Molster CM, Bowman FL, Bilkey GA, Cho AS, Burns BL, Nowak KJ (2018). The evolution of public health genomics: exploring its past, present, and future. Front Public Health.

[26] Japanese Nursing Association. Nursing in Japan. Japan Nursing Association. https://www.nurse.or.jp/jna/english/nursing/system.html. Accessed 10 Dec 2019.

[27] Sharoff L (2015). Genetics and genomics integration into undergraduate nursing education. J Nurs Educ Pract.

[28] Wilson LO (2016). Three domains of learning – cognitive, affective, psychomotor.

[29] Kawasaki H, Rahman MM, Iwasa M, Kukinaka C (2020). Effectiveness of a basic education program on radiation-related health concerns for nurses of public health and school health in Japan. Iran J Public Health.

[30] Keller JM. Development and use of the ARCS model of instructional design. J Instr Dev. 1987. 10.1007/BF02905780.

[31] Kanatani Y, Tomita N, Sato Y, Eto A, Omoe H, Mizushima H (2017). National Registry of designated intractable diseases in Japan: present status and future prospects. Neurolog Medico-Chirurg.

[32] Junyent M, Gilabert R, Zambón D, Pocoví M, Mallén M, Cofán M, et al. Femoral atherosclerosis in heterozygous familial hypercholesterolemia: influence of the genetic defect. Arterioscler Thromb Vasc Biol. 10.1161/ATVBAHA.107.153841. 10.1161/ATVBAHA.107.153841 18096825

[33] R Core Team (2020). R: A language and environment for statistical computing.

[34] Clark V, Braun V (2013). Teaching thematic analysis: overcoming challenges and developing strategies for effective learning. Psychologist.

[35] Lunyera J, Kirenga B, Stanifer JW, Kasozi S, van der Molen T, Katagira W (2018). Geographic differences in the prevalence of hypertension in Uganda: results of a national epidemiological study. PLoS One.

[36] Raeisi A, Mehboudi M, Darabi H, Nabipour I, Larijani B, Mehrdad N (2017). Socioeconomic inequality of overweight and obesity of the elderly in Iran: Bushehr elderly health (BEH) program. BMC Public Health.

[37] Congdon P (2019). Obesity and urban environments. Int J Environ Res Public Health.

[38] Tonkin E, Calzone K, Jenkins J, Lea D, Prows C (2011). Genomic education resources for nursing faculty. J Nurs Scholarsh.

[39] Paneque M, Turchetti D, Jackson L, Lunt P, Houwink E, Skirton H (2016). A systematic review of interventions to provide genetics education for primary care. BMC Fam Pract.

[40] Horii S, Okuda H, Kawasaki C, Osawa E, Morinaga Y, Naruki H (2019). Competencies and the related factors of senior administrative public health nurses: a study based on the “standardized career ladder.” [Nihon koshu eisei zasshi]. Jpn J Public Health.

[41] Jackson L, O'Connor A, Paneque M, Curtisova V, Lunt PW, Pourova RK (2019). Correction: The Gen-Equip Project: evaluation and impact of genetics e-learning resources for primary care in six European languages. Genet Med.

[42] Merikangas KR, Risch N (2003). Genomic priorities and public health. Science.

[43] Tan PY, Mitra SR, Amini F (2018). Lifestyle interventions for weight control modified by genetic variation: a review of the evidence. Public Health Genom..

[44] Bowman FL, Molster CM, Lister KJ, Bauskis AT, Garton-Smith J, Vickery AW (2019). Identifying perceptions and preferences of the general public concerning universal screening of children for familial hypercholesterolaemia. Public Health Genom.

[45] Kent S, Bardach SH, Zhang X, Abner EL, Grill JD, Jicha GA (2018). Public understanding and opinions regarding genetic research on Alzheimer’s disease. Public Health Genom..

[46] Kudo Y, Hayashi S, Yoshimura E, Tsunoda M, Tsutsumi A, Shibuya A (2013). Five reasons for the lack of nursing students’ motivation to learn public health. Tohoku J Exp Med.

[47] Kofi Aduo-Adjei K, Emmanuel O, Forster O (2016). The impact of motivation on the work performance of health workers (Korle Bu Teaching Hospital): Evidence from Ghana. Hosp Pract Res.

[48] Asadi N, Memarian R, Vanaki Z (2019). Motivation to care: a qualitative study on Iranian nurses. J Nurs Res.

